# Supplementary material for: Metabarcoding analysis reveals hidden eukaryotic plankton biodiversity in the Ross Sea, Antarctica
Source: PeerJ. 2025 Oct 14;13:e20118. doi: 10.7717/peerj.20118 (PMC12533539; doi:10.7717/peerj.20118)
Supplement: Supplemental Information 1 — Temp: temperature (°C); Sal: salinity (PSU); DO: dissolved oxygen (umol/kg) [file peerj-13-20118-s001.docx]

**Table S1:**

Results of the environmental parameters measured in the Ross Sea sampling region.
Temp: temperature (°C); Sal: salinity (PSU); DO: dissolved oxygen (umol/kg)

| **Site** | **Depth (m)** | **Temp (°C )** | **Sal (PSU)** | **DO (umol/kg)** |  | **Site** | **Depth (m)** | **Temp (°C )** | **Sal (PSU)** | **DO (umol/kg)** |
| --- | --- | --- | --- | --- | --- | --- | --- | --- | --- | --- |
|  | 271 | -1.91 | 34.78 | 303.03 |  |  | 300 | -1.45 | 34.57 | 285.59 |
| 12 | 100 | -1.68 | 34.77 | 322.11 |  | 46 | 150 | -1.15 | 34.51 | 276.44 |
|  | 0 | 0.85 | 34.52 | 403.31 |  |  | 0 | -1.02 | 34.17 | 351.75 |
|  | 491 | -1.98 | 34.79 | 307.24 |  |  | 400 | -1.83 | 34.64 | 290.36 |
| 16 | 200 | -1.92 | 34.76 | 301.33 |  | 50 | 120 | -1.34 | 34.46 | 276.86 |
|  | 0 | 0.75 | 33.80 | 404.21 |  |  | 0 | -0.95 | 34.19 | 351.65 |
|  | 1000 | -1.89 | 34.89 | 315.48 |  |  | 330 | -1.89 | 34.66 | 294.73 |
| 18 | 200 | -1.89 | 34.77 | 307.63 |  | 52 | 60 | -1.07 | 34.44 | 339.88 |
|  | 0 | -0.33 | 33.83 | 393.16 |  |  | 0 | -0.44 | 34.24 | 366.83 |
|  | 300 | -1.90 | 34.76 | 298.50 |  |  | 2000 | 0.647 | 34.70 | 227.29 |
| 26 | 100 | -1.88 | 34.67 | 294.88 |  | 67 | 40 | -1.01 | 33.92 | 349.11 |
|  | 0 | -1.52 | 33.56 | 365.48 |  |  | 0 | -0.74 | 33.86 | 357.18 |
|  | 573 | -1.89 | 34.78 | 299.64 |  |  | 2638 | 0.04 | 34.67 | 252.85 |
| 29 | 150 | -1.51 | 34.57 | 277.12 |  | 69 | 200 | -0.62 | 34.42 | 256.99 |
|  | 0 | 0.03 | 34.19 | 385.26 |  |  | 0 | -1.24 | 33.72 | 369.67 |
|  | 600 | -1.90 | 34.77 | 304.82 |  |  | 2596 | 0.50 | 34.70 | 233.97 |
| 35 | 180 | -2.04 | 34.57 | 305.75 |  | 71 | 200 | -1.79 | 34.17 | 294.89 |
|  | 0 | -0.48 | 34.30 | 370.30 |  |  | 0 | -0.37 | 33.76 | 391.32 |
|  | 500 | -1.89 | 34.78 | 299.43 |  |  | 565 | -1.07 | 34.47 | 273.02 |
| 38 | 100 | -1.37 | 34.54 | 276.93 |  | 77 | 200 | -1.62 | 34.23 | 300.72 |
|  | 0 | -0.09 | 34.28 | 368.47 |  |  | 0 | -0.99 | 34.08 | 351.38 |
|  | 570 | -1.89 | 34.77 | 298.75 |  |  | 532 | -1.84 | 34.47 | 298.41 |
| 44 | 200 | -1.70 | 34.63 | 286.24 |  | 80 | 200 | -1.77 | 34.41 | 294.12 |
|  | 0 | -0.73 | 34.14 | 357.19 |  |  | 0 | -0.51 | 33.99 | 378.37 |
