# Supplementary material for: Metabarcoding analysis reveals hidden eukaryotic plankton biodiversity in the Ross Sea, Antarctica
Source: PeerJ. 2025 Oct 14;13:e20118. doi: 10.7717/peerj.20118 (PMC12533539; doi:10.7717/peerj.20118)
Supplement: Supplemental Information 2 [file peerj-13-20118-s002.docx]

**Table S2:
Sequencing and bioinformatic analysis results information of each sample.**

| **Site** | **Depth** | **Number of OTUs** | **Number of Reads** |  |  | **Site** | **Depth** | **Number of OTUs** | **Number of Reads** |
| --- | --- | --- | --- | --- | --- | --- | --- | --- | --- |
|  | 271 | 4,669 | 259,893 |  |  |  | 300 | 3,405 | 241,969 |
| 12 | 100 | 3,767 | 228,999 |  |  | 46 | 150 | 3,651 | 249,967 |
|  | 0 | 5,154 | 279,836 |  |  |  | 0 | 4,265 | 261,669 |
|  | 491 | 3,165 | 227,742 |  |  |  | 400 | 2,078 | 156,216 |
| 16 | 200 | 3,191 | 239,383 |  |  | 50 | 120 | 4,033 | 269,434 |
|  | 0 | 3,828 | 221,371 |  |  |  | 0 | 4,285 | 254,598 |
|  | 1000 | 2,226 | 213,244 |  |  |  | 330 | 3,842 | 248,779 |
| 18 | 200 | 2,707 | 202,370 |  |  | 52 | 60 | 4,300 | 253,118 |
|  | 0 | 4,180 | 246,264 |  |  |  | 0 | 3,470 | 262,961 |
|  | 300 | 3,442 | 260,660 |  |  |  | 2000 | 4,433 | 287,965 |
| 26 | 100 | 3,713 | 266,157 |  |  | 67 | 40 | 4,073 | 265,143 |
|  | 0 | 4,417 | 260,050 |  |  |  | 0 | 4,757 | 279,679 |
|  | 573 | 3,572 | 264,707 |  |  |  | 2638 | 2,937 | 219,533 |
| 29 | 150 | 3,580 | 256,597 |  |  | 69 | 200 | 3,181 | 238,528 |
|  | 0 | 4,246 | 257,020 |  |  |  | 0 | 5,346 | 243,606 |
|  | 600 | 1,971 | 177,673 |  |  |  | 2596 | 2,207 | 167,318 |
| 35 | 180 | 4,458 | 278,899 |  |  | 71 | 200 | 1,229 | 215,817 |
|  | 0 | 3,741 | 229,204 |  |  |  | 0 | 3,316 | 218,230 |
|  | 500 | 2,839 | 253,431 |  |  |  | 565 | 1,731 | 202,978 |
| 38 | 100 | 3,964 | 232,427 |  |  | 77 | 200 | 2,575 | 201,251 |
|  | 0 | 4,378 | 257,152 |  |  |  | 0 | 4,679 | 246,446 |
|  | 570 | 3,113 | 273,311 |  |  |  | 532 | 2,289 | 218,644 |
| 44 | 200 | 2,906 | 263,297 |  |  | 80 | 200 | 3,025 | 214,063 |
|  | 0 | 4,856 | 284,154 |  |  |  | 0 | 4,789 | 236,943 |
