# Supplementary material for: Metabarcoding analysis reveals hidden eukaryotic plankton biodiversity in the Ross Sea, Antarctica
Source: PeerJ. 2025 Oct 14;13:e20118. doi: 10.7717/peerj.20118 (PMC12533539; doi:10.7717/peerj.20118)
Supplement: Supplemental Information 3 — The percentage in parentheses indicates the proportions relative to the total number of OTUs and reads, respectively. [file peerj-13-20118-s003.docx]

**Table S3:**
**Number of OTUs and reads assigned for each taxonomical classification level**

The percentage in parentheses indicates the proportions relative to the total number of OTUs and reads, respectively.

| **Taxonomic Level** | **Number of OTUs** | **Number of Reads** |
| --- | --- | --- |
| **Unassigned** | 250 (2.68 %) | 804 (0.09 %) |
| **Kingdom** | 71,332 (99.65 %) | 4,832,614 (99.98 %) |
| **Phylum** | 54,723 (76.45 %) | 4,570,796 (94.57 %) |
| **Class** | 53,626 (74.92 %) | 4,514,881 (93.41 %) |
| **Order** | 19,683 (27.50 %) | 2,012,753 (41.64 %) |
| **Family** | 17,624 (24.62 %) | 1,970,666 (40.77 %) |
| **Genus** | 15,765 (22.02 %) | 1,514,487 (31.33 %) |
| **Species** | 9,338 (13.05 %) | 930,950 (19.26 %) |
