# Supplementary figures and images for: Metabarcoding analysis reveals hidden eukaryotic plankton biodiversity in the Ross Sea, Antarctica
Source: PeerJ. 2025 Oct 14;13:e20118. doi: 10.7717/peerj.20118 (PMC12533539; doi:10.7717/peerj.20118)

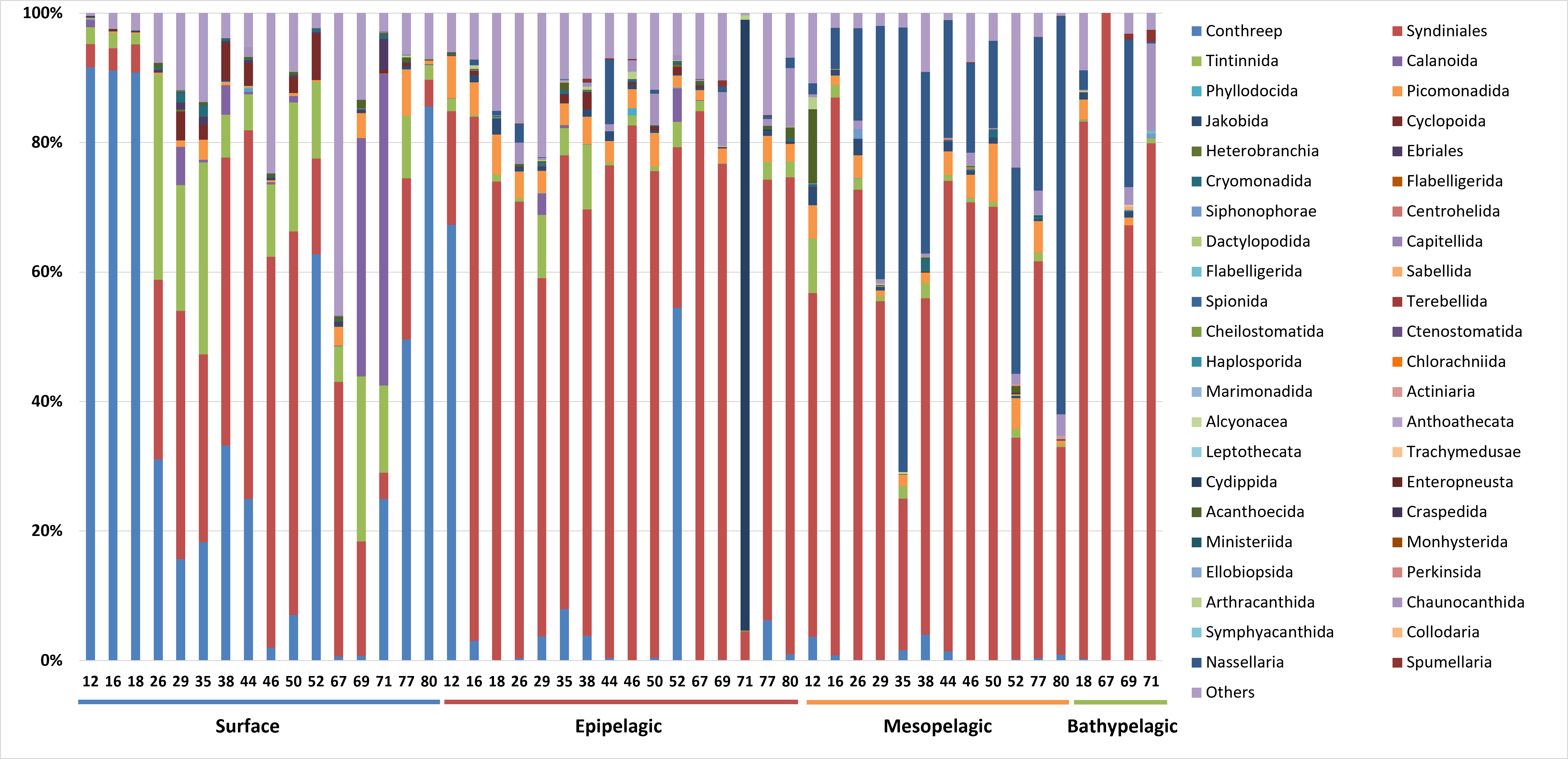

Supplement: Supplemental Information 4 — The samples of each site were mainly composed of 44 orders of zooplankton. [file peerj-13-20118-s004.png]
